# Identifying factors associated with experiences of coronary heart disease patients receiving structured chronic care and counselling in European primary care

**DOI:** 10.1186/1472-6963-12-221

**Published:** 2012-07-27

**Authors:** Sabine Ludt, Jan van Lieshout, Stephen M Campbell, J Rochon, Dominik Ose, T Freund, Michel Wensing, Joachim Szecsenyi

**Affiliations:** 1 Department of General Practice and Health Services Research, University Hospital of Heidelberg, Voßstrasse 2, 69115, Heidelberg, Germany; 2 Scientific Institute for Quality of Healthcare, Radboud University Nijmegen Medical Centre, P.O. Box 9101, 6500, HB, Nijmegen, The Netherlands; 3 Health Sciences – Primary Care Group, University of Manchester, Williamson Building, Manchester, M13 9PL, UK; 4 Institute of Medical Biometry and Informatics, University of Heidelberg, Im Neuenheimer Feld 305, 69120, Heidelberg, Germany

## Abstract

**Background:**

Primary care for chronic illness varies across European healthcare systems. In patients suffering from coronary heart disease (CHD), factors associated with patients’ experiences of receiving structured chronic care and counselling at the patient and practice level were investigated.

**Methods:**

In an observational study comprising 140 general practices from five European countries (Austria, Germany, the Netherlands, Switzerland and the United Kingdom), 30 patients with Coronary Heart Disease (CHD) per practice were chosen at random to partake in this research. Patients were provided with a questionnaire and the Patient Assessment of Chronic Illness Care (PACIC-5A) - instrument. Practice characteristics were assessed through a practice questionnaire and face to face interviews. Data were aggregated to obtain two practice scores representing quality management and CHD care, respectively. A hierarchical multilevel analysis was performed to examine the impact of patient and practice characteristics on PACIC scores.

**Results:**

The final sample included 1745 CHD-patients from 131 general practices with a mean age of 67.8 (SD 9.9) years. The overall PACIC score was 2.84 (95%CI: 2.79; 2.89) and the 5A score reflecting structured lifestyle counselling was 2.75 (95% CI: 2.69; 2.79). At the patient level, male gender, more frequent practice contact and fewer related or unrelated conditions were associated with higher PACIC scores. At the practice level, performance scores reflecting quality management (*p = 0.013*) and CHD care (*p = 0.009*) were associated with improved assessment of the structured chronic care and counselling received.

**Conclusions:**

Patients’ perceived quality of care varies. However, good practice management and organisation of care were positively reflected in patients’ assessments of receiving structured chronic illness care. This highlights the importance of integrating patient experiences into quality measurements to provide feedback to health care professionals.

## Background

Cardiovascular disease (CVD) and notably coronary heart disease (CHD) are important causes of morbidity, contributing substantially to escalating healthcare costs [1]. Cardiovascular risk management (CVRM), the majority of which is provided in primary care, includes counselling on lifestyle, preventive medication and continuous monitoring [2,3]. Not all patients suffering from CVD receive optimal care [4,5], which may be related to a range of factors including practice characteristics, and the structure of the care and counselling, which differs considerably across Europe [6].

### Chronic Care Model (CCM)

The Chronic Care Model (CCM) is a widely adopted framework to enhance evidence based chronic care [7,8]. It describes a proactive patient centred care approach that is planned and includes goal setting, problem-solving and follow-up support. The principles of the CCM are included in disease management programs in various health-care systems. Previous research demonstrated that elements of the CCM have been associated with improved quality of care and patient outcomes [9,10]. Furthermore, there is evidence that a strong primary health-care orientation, with general practitioners acting as gatekeepers providing the first contact with patients within the health care system and ensuring continuity of care, is linked to the adoption of the CCM [11] and improved chronic illness care and outcomes [12].

### 5A Approach of behaviour change counselling

The 5A approach concerns behaviour change counselling, which is integral to the CCM, and provides a sequence of evidence-based brief intervention steps (Assess, Advise, Agree, Assist and Arrange). These steps are recommended for behavioural counselling and self-management support in primary care settings to address a broad range of behaviours and health conditions [13].

### The Patient Assessment of Chronic Illness Care (PACIC)- General Plus 5A

The Patient Assessment of Chronic Illness Care (PACIC) questionnaire [14,15] allows patients to assess whether the care they have received is congruent to the principles of the CCM. The 20-item PACIC questionnaire was developed and validated by Glasgow et al [14] and was later revised to include six new items assessing self-management support according to the 5A approach [16]. The instrument has been translated, validated and used by patients suffering from various conditions in several countries [17-20]. Recently, the PACIC was used to evaluate case -and disease-management interventions [21,22].

### Quality measurement in primary care

Since 2001, attempts to assess and improve the quality of primary care have led to the development of two instruments as part of the “European Task Force for Methods of Assessment, Quality Management and Certification in Health Care” (TOPAS Europe)^1^ research project. These instruments are based on quality indicators, and were developed using validated systematic consensus techniques, expert panels and empirical testing [23]. The European Practice Assessment (EPA)- practice management instrument was developed to measure the quality of practice management [24,25]. During the autumn of 2003, the EPA practice management instrument came into operation in nine countries [24]. EPA’s key aspects of activity are the development and validation of a set of indicators and tools describing the organisational aspects of primary care practices. The EPA indicators were incorporated into questionnaires, interview guides and check-lists based on an extended review of the international literature concerning assessment models for primary care, with special attention being paid to the Dutch model of practice visits. The indicator-based instrument has been demonstrated to provide valid, reliable and feasible results for quality improvement in primary health care [25]. Since 2005 this instrument has been revised every three years, and this cycle will continue, as the instrument is used for benchmarking ambulatory care practices [26]. The second instrument developed was the EPA-Cardio instrument, which assesses the quality of cardiovascular care and risk management [27,28], and was developed between 2006 and 2008 [28].

### Quality measurement from the patients’ perspective

Quality of care is multidimensional and there are various aspects of quality and several methods relating to quality measurement [29,30]. Patient involvement and engagement are imperative to achieving good outcomes during chronic care [7], and patient experience is an integral part of quality measurement and improvement [31]. Recently, international studies have demonstrated a poor association between practice characteristics and patient satisfaction [32-34]. However, the performance of health care providers may not be reflected in patients’ assessments of quality if general summarising satisfaction scores are used [34]. Reports of patient experience are increasingly replacing assessments of patient satisfaction, as they can highlight processes of care in detail including providing self-management support, and can be helpful for providing feedback to health care professionals [35].

Therefore, the aim of this international observational study, conducted as part of the development of the EPA-Cardio instrument, was to estimate patients’ experience of receiving structured chronic care and counselling, and to examine the extent to which quality processes reported in general practices and individual patient characteristics were associated with patients’ experience of structured chronic care and counselling. As the PACIC instrument, our primary outcome, questions care processes received by patients within a six month time period, we hypothesised that this process-oriented instrument would be associated with practice reported process measures rather than outcome-oriented instruments used to assess patient satisfaction.

## Methods

### Setting

This study was part of the European Practice assessment (EPA) - Cardio project, which focused on the assessment of cardiovascular prevention and management in European primary care. In the first stage of the 4-year EPA-Cardio project (which began in 2006) we developed quality indicators to measure cardiovascular prevention and care [27], and identified measures for use in a subsequent observational study [28]. The international cross-sectional observational study was conducted in 10 European countries between 2008 and 2009, i.e. Austria, Belgium, Finland, France, Germany, the Netherlands, Slovenia, Spain, Switzerland and the United Kingdom [36]. As the English, Dutch and German versions of PACIC - instrument were validated; only the UK, Germany, Austria, Switzerland and the Netherlands data were included in this study. Ethics committees in all participating countries approved the study.

### Recruitment of participants

The recruitment of countries, practices and patients has been described in detail elsewhere [36]: In summary, general practices were randomly sampled by each national research team according to the national distribution of general practices related to practice size and location, with the intention of recruiting a representative sample of 36 practices per country.

We included patients with coronary heart disease (CHD), e.g. myocardial infarction, angina pectoris or vascular surgery, who were identified from the practice records by the presence of a diagnosis code or active current medication. Patients with terminal illness or significant cognitive impairments were excluded. Of the eligible patients, a sample of 30 patients per practice was randomly selected to be posted a questionnaire, with an expected attrition rate of 50%.

### Measures

#### Patient level

The patient questionnaire included demographic items (e.g. education), two questions derived from the quality indicators developed during the first phase of the project [27], and the PACIC-5A instrument (Additional file 1) as the main outcome measure. PACIC is a validated measure of patients’ perception of chronic illness care that questions elements of care and counselling received by patients. We used the 26-item instrument that asked patients to indicate how often they received a care element within the last six months, e.g. “being asked how chronic illness affects one’s life”. Each item can be answered with “almost never”, “generally not” “sometimes”, “most of the time” or “almost always”, with a score of one for the first answer (almost never) up to five (almost always). The item scores aggregate into five subscales that align with components of the CCM. In addition, there are subscales for the five steps of the 5A counselling model (Assess, Advise, Agree, Assist, and Arrange).

Two quality indicators that were developed during the first stage of the EPA Cardio project were included in the questionnaire [28]: “All patients with established CVD should be offered referral to a supervised cardiac exercise rehabilitation program (e.g. referred by either GP or hospital specialist)” and “All patients with established CVD should be asked about the quality of care they receive from their practice”. These indicators were paired to questions that could be answered with “yes”, “no” or “don’t know”.

#### Practice level

Researchers collated data concerning practice characteristics and quality measures through practice questionnaires and face-to-face or telephone interviews with leading GPs using standardized interview guides. These instruments contained questions to characterise the practice according to size, location and practice staff, and quality indicators that were developed during the EPA-Cardio project [27] and those derived from the EPA practice management instrument [24,25]. The quality indicators covered CVD care (33 indicators) [27] and organizational aspects of the practice management in the three dimensions of ‘information process and technology’ (11 indicators), ‘organization of chronic care and prevention’ (19 indicators) and ‘quality improvement’ (13 indicators) [24]. To score practice quality indicators, items of the practice questionnaires were aggregated using the homogeneity analysis by alternating least squares (HOMALS). With this factor analysis, 32 binary items with discrimination measures over 0.4 were identified, loading on two dimensions “quality management” (15 items) and “CVD care (17 items) (Table 1). Scores were calculated through summation of the number of ‘yes’- answers, resulting in a range from 0 to 15 for the quality-management score, and from 0 to 17 for the CVD-care score.

**Table 1:** Practice quality indicators

	**Quality dimension**	
**Items**	**Quality management**	**CVD care**
1	Does the practice use a computer-supported patient file system?	Does the practice use case finding methods to detect patients with cardiovascular risk factors?
2	Is the computer used for creating medication prescriptions?	Does the practice use a system for recalling patients with cardio vascular diseases?
3	Does the practice have a procedure for the management of patient information in relation to detailed examination results and the documentation of measures that were taken (e.g., blood examinations)?	Does the practice use a system for recalling patients with diabetes?
4	Does the practice have a procedure for the management of patient information in relation to the review of detailed examination results by the doctor (in terms of outgoing needs)?	Does the practice use a system for recalling patients with hypertension?
5	Do the practice doctors have direct access to medical guidelines (either on paper or electronic) in their treatment rooms?	Does the practice use a system for recalling populations at risk for preventive care regarding cardio vascular diseases?
6	In general: Is practice staff allowed to contact or recall patients?	Does the practice use a system for recalling populations at risk for preventive care regarding influenza?
7	Does the practice produce a quality report?	Does the practice have a procedure for smoking cessation (e.g. with the Minimal Intervention Strategy)
8	Has the practice undertaken at least one clinical audit in the last 12 months?	Does the practice participate in public health care programs on life style (physical exercise, stop smoking)?
9	Did you set standards regarding this clinical audit (defined the target)?	Did all nurses attend ≥ one training/continuing medical education event on CVD within the last 5 years?
10	Did you collect data regarding this clinical audit?	Did nurses take part in local/community campaigns or actions on CVD risk prevention (e.g. stop smoking campaigns, fun-runs etc.)?
11	Did you evaluate the result?	Does the practice use a CVD standardized risk assessment tool?
12	Were you able to improve the quality regarding this clinical audit topic?	Is the CVD risk assessment tool integrated with the patient medical record system (e.g. so that the CVD event risk score is entered directly in to the patient's medical record)
13	Does the practice have a critical incident register?	Is there in general a record in the electronic or paper based patient record that the CVD standardized risk assessment tool has been offered?
14	Did the practice have a team meeting about quality improvement relating to CVD at least once in the last 15 months?	Is CVD risk advice (e.g. about modifiable risk factors such as diet and exercise) integrated with the patient medical record system?
15	Did the practice participate in cardiovascular quality improvement projects?	Do you offer regularly two or many consultations to provide advice on patient’s life style?
16		Does the practice have an up-to-date directory of prevention activities/organizations available locally (e.g. gyms, walking group, weight-watchers etc.)?
17		Did your practice participate in a project concerning cardiovascular risk management the last 2 years (apart from those mentioned above)?

All measures were piloted before being used during the study [25,28].

### Analyses

The main outcome measure was the overall PACIC score, ranging between one and five, with higher scores indicating better patient perceived quality of chronic illness care. We calculated mean overall PACIC scores, overall 5A scores and subscale scores, following the scoring instructions of Glasgow et al (Additional file 1) [16].

Due to the hierarchical structure of the data, multilevel analysis was applied, which takes into account the non-independence of patient observations (level 1) nested within practices (level 2), and these nested within countries (level 3). Several models were evaluated:

Multilevel linear analysis began with a three-level null (empty) model with no predictor variables in the fixed part and only the intercepts in the random part of the model (M1). This model can be used as a reference for comparing the size of contextual (practice or country) variations in PACIC scores in subsequent models.

The next model (M2) included patient-level variables as fixed effects only. Finally, explanatory variables at the patient and practice level were added as fixed effects resulting in the best model fit (M3). Variance partition coefficients at each level were calculated using the restricted maximum likelihood (REML). The random-part results of the null model (M1) are reported together with the corresponding intra-class correlations (ICC) at the practice and country level [37]. Finally, the fixed-part results of the full 3-level random-intercept model (M3) are presented. Variance partition coefficients with corresponding two-sided 95% confidence intervals (CI) are provided.

The significance level was set to 5% (two-sided). All statistical analyses were carried out using SPSS version 18.0 (SPSS Inc., Chicago, IL, USA), with the exception of the multilevel analysis. This analysis was conducted by using the procedure PROC MIXED in SAS version 9.2 (SAS Institute, Cary, NC).

## Results

### Practice and Patient Characteristics

The total sample comprised 2152 patients with CHD in 140 primary care practices in five countries: Austria (28 practices), Germany (24 practices), the Netherlands (34 practices), Switzerland (22 practices) and the UK (32 practices). Four practices (16 patients) were excluded as they had enrolled fewer than five patients. For 1958 individuals (91%), PACIC sum scores could be calculated. Only patients with complete data concerning all explanatory variables were considered in the final model of the multilevel hierarchical analysis, reducing the sample size to 1745 patients from 131 practices (81.1%). A non-responder analysis demonstrated no clinically important differences. On average, excluded patients were 1.7 years older, had less yearly practice contacts and slightly less medical conditions, compared with patients included in analyses (Table 2). Of the final patient sample, 31.6% were female and the mean age was 67.8 years (SD 9.9). The majority of patients had been in school for more than nine years (68.7%) and had been visiting their practices for more than seven years (83.7%). Approximately 75% of the sample had never been asked to assess the quality of care of their practice previously. On average, patients self-reported as having three medical conditions (Table 2).

**Table 2:** Practice (n=140) and patient characteristics (n=2152)

	**included (n=1745)**		**not included (n=407)**		***P***^***1***^
**Patient level**					
**Age (years):** mean (SD)	67.8	(9.9)	69.5	(9.4)	0.002
**Gender**					0.807
Female % (n)	31.6	552	3.9	116	
Male % (n)	68.4	1193	69.1	260	
**Education**^**2**^					0.489
<= 9 years % (n)	31.3	(547)	33.2	(116)	
> 9 years % (n)	68.7	(1198)	66.8	(233)	
**Frequency of practice attendance**^**3**^					0.028
<= 3 times / year % (n)	34.2	(596)	41.5	(149)	
4-7 times/ year % (n)	38.7	(675)	33.7	(121)	
> 7 times / year % (n)	22.5	(474)	24.8	(89)	
**Duration of being patient in practice**^**4**^					0.415
up to 2 years % (n)	3.7	(64)	3.8	(14)	
3-7 years % (n)	12.7	(221)	1.2	(38)	
> 7 years % (n)	83.7	(1460)	86.1	(321)	
**Already been asked for quality assessment**^**5**^					0.099
No % (n)	75.5	(1317)	78.7	(266)	
Don’t know % (n)	9.8	(171)	1.9	(37)	
Yes % (n)	14.7	(257)	1.4	(35)	
**Number of conditions (range: 0–11)**^**6**^**;** mean (SD)	3.3	(1.7)	3.0	(1.8)	0.000
**Practice level**					
**N** (n per practice; range)	131	(13; 6–37)	9	(10; 4–25)	
**FTE**^**7**^**GP:** mean (SD)	1.83	(1.57)	1.95	(1.69)	0.233
**Quality management (range: 0–15)**^**8**^ mean (SD)	9.84	(3.82)	9.42	(3.99)	0.063
**CVD care (range: 0–17)**^**8**^ mean (SD)	8.54	(4.71)	8.84	(4.38)	0.228

^1^ p values are based on χ^2^ tests for categorical variables and on t tests for continuous variables.

^2^ Questionnaire: How many years did you stay at school? < 9 years; 10 – 13 years; > 13 years.

^3^ Questionnaire: How often do you usually attend your GP within 12 months? 0–1 times; 2–3 times; 4–5 times; 6–7 times; 8–9 times; more than 10 times.

^4^ Questionnaire: How long have you been a patient with your practice? Less than 1 year; 1–2 years; 3–7 years; 8–12 years; more than 13 years.

^5^ Questionnaire: Have you **ever** been asked about the quality of care you receive from your practice (e.g. by questionnaire)? Yes, no, don’t know.

^6^ Questionnaire: Do you have any one or more of the following diseases or conditions? High blood pressure, Hypercholesterinaemia (high cholesterol), Diabetes, Angina, Heart attack (myocardial infarction), Coronary surgery / PCTA (Percutaneous Transluminal Angioplasty), Heart failure, Transient ischaemic attack (TIA), Stroke, Peripheral artery disease (PAD), Depression – yes, no, don’t know; Yes answers - theoretical range: 0–11; observed range:0–10.

^7^ FTE= Full time equivalent GP.

^8^ Theoretical and observed range (Table 1).

The mean number of patients included per practice was 13, ranging from 6-37. The mean full time equivalent GPs reported working at each practice was 1.8 (SD 1.6). The mean practices’ scores were 9.8 (SD 3.8) for “quality management”, ranging from 0-17, and 8.5 (SD 4.7) for “CVD care” (range: 0-15). The included practices (n = 131) did not differ from the excluded practices (n = 9) in relation to any variables.

### PACIC and 5A scores

The mean overall PACIC score (range 1-5), reflecting elements of the chronic care model (CCM), was 2.84 [CI: 2.79; 2.89]. The mean 5A score (range 1-5), reflecting structured behaviour change counselling, was 2.75 [CI: 2.69; 2.79]. Mean sum scores and subscale scores differed from one another, with higher scores for chronic care processes such as ‘patient activation’ than for counselling steps such as ‘assess’ or ‘advise’. Highest scores were calculated for the subscale ‘delivery system/practice design, i.e. 3.35 [95% CI: 3.30; 3.40], representing organizational aspects of care. Lowest scores were found in the subscales on follow up management reflecting continuity of care: The subscale ‘Follow up/coordination’ scored 2.51 [95% CI: 2.46; 2.57] and the subscale ‘arrange’ achieved 2.38 [95% CI: 2.32; 2.43] (Table 3).

**Table 3:** Patient Assessment of Chronic Illness Care (PACIC)

	**Mean**	**Standard error**	**[95% confidence interval]**	**N**
**Overall PACIC score**	2.84	0.03	[2.79; 2.89]	1958
Patient activation	3.10	0.03	[3.04; 3.16]	1962
Delivery system/practice design	3.35	0.03	[3.30; 3.40]	1905
Goal setting/tailoring	2.63	0.03	[2.58; 2.69]	1921
Problem solving/contextual	2.88	0.03	[2.82; 2.94]	1905
Follow up/coordination	2.51	0.03	[2.46; 2.57]	1889
**Overall 5A counseling score**	2.75	0.03	[2.70; 2.79]	1892
Assess	2.88	0.03	[2.82; 2.93]	1926
Advise	2.87	0.03	[2.82; 2.92]	1934
Agree	2.98	0.03	[2.92; 3.03]	1932
Assist	2.58	0.03	[2.52; 2.63]	1906
Arrange	2.38	0.03	[2.32; 2.43]	1882

### Factors associated with patients’ assessment of receiving structured chronic care and counselling

#### Random part results

The random-part results indicated that variation in the PACIC scores was predominantly due to variations in individual characteristics or differences between countries, rather than differences between practices. The total variance in the null model was 1.235. The proportion of variance (ICC) was 2.7% at the practice level and 26.0% at the country level. The residual proportion of variance including variance at patient level plus random was 71%. Including explanatory variables at patient and practice level explained the variance between countries to 10% and between practices to 22%.

#### Fixed part results

Table 4 presents the results for the overall PACIC score: Adjusted for all other variables included in the model, age and educational level were not significantly associated with patients’ assessment of receiving structured care and counselling (PACIC score). Being female was associated with a 0.06 point lower PACIC score compared with males (*p = 0.012*). Higher PACIC scores (0.22) were associated with patients who had been visiting their practice for less than two years compared with the reference category of visiting a practice for more than seven years (*p = 0.001*). Attending a practice three times or fewer per year was associated with lower PACIC scores (-0.16; *p = 0.007*) compared with attending more than seven times per year. Lower PACIC scores were achieved if patients were asked to assess the received care for the first time (-0.48) compared with patients that had been asked to assess this previously (*p = 0.000)*. Patients’ assessment was associated with the number of medical conditions, resulting in a 0.01 decrease of the PACIC score with each additional condition (*p = 0.008)*.

**Table 4:** Parameter estimates of the final multilevel model with overall PACIC score as dependent variable(N = 1745 patients, 131 practices, 5 countries)

	**Regression coefficient**	**Standard error**	**[95% confidence interval]**	***P- value***
**PATIENT CHARACTERISTICS**				
**Age**^**1**^				
Continuous (5 years)	−0.00	0.02	[−0.03; 0.03]	*0.819*
**Gender**^**2**^				
Female	−0.06	0.03	[−0.11; -0.14]	*0.012*
Male			Reference	
**Education**				
≤ 9 years	0.03	0.06	[−0.10; 0.15]	*0.678*
> 9 years			Reference	
**Frequency of practice attendance**				
Up to 3 times per year	−0.16	0.06	[−0.427 -0.04]	*0.007*
4–7 times per year	−0.11	0.06	[−0.13; 0.11]	*0.842*
more than 7 times per year s			Reference	
**Duration of being patient in practice**				
Up to 2 years	0.22	0.07	[0.09; 0.36]	*0.001*
3–7 years	−0.04	0.05	[−0.14; 0.07]	*0.509*
more than 7 years			Reference	
**Have you ever been asked about the quality of care you receive from your practice (e.g. by questionnaire)**				
No	−0.48	0.06	[−0.61; -0.36]	*0.000*
Don’t know	−0.03	0.04	[−0.11; 0.06]	*0.533*
Yes			Reference	
**Number of conditions**				
Continuous (0–11)	−0.01	0.01	[−0.03; -0.00]	*0.008*
**PRACTICE CHARACTERISTICS**				
**Full time equivalents (FTE) GPs**				
Continuous	−0.02	0.02	[−0.06; 0.02]	*0.328*
**Practice quality management**				
Continuous	0.02	0.01	[0.01; 0.03]	*0.013*
**Practice CVD care**				
Continuous	0.01	0.00	[0.01; 0.02]	*0.009*

^1^ For continuous variables regression coefficients indicate the change of the overall PACIC score with each increasing unit of this variable.

^2^ For categorical variables regression coefficients indicate the changes of the overall PACIC score in comparison to a reference category.

At the practice level, “CVD care” (0.01) and “quality management” (0.02) scores were associated with increasing PACIC scores. The regression coefficient indicates that for instance, each additional self-reported quality item of the CVD care score results in a 0.01 increase in the PACIC score. As practices included in the sample achieved the whole range of quality items from 0 to 17 (Table 1), the difference between the PACIC scores of practices with the lowest scores compared with those with the highest scores was 17 × 0.01 = 0.17. The coefficients for the quality management score require comparable interpretation, resulting in a maximum difference of 15 × 0.02 = 0.3 points in PACIC scores. The number of full time equivalent GPs, reflecting the practice size, was not significantly associated with PACIC scores.

## Discussion

This study has three main findings: Firstly, patients with coronary heart disease from European general practices perceive that the quality structured chronic care and counselling is not optimal. During this research the mean overall PACIC score was calculated as 2.84 (maximum = 5). During previous research, the PACIC instrument has been applied to a wide range of chronic conditions and populations [38,39] including individuals suffering from diabetes [21], coronary heart disease (CHD) [17], osteoarthritis [18] and mental health [19], with overall scores reported being between 2.49 [18] and 3.80. Compared to diabetes care [16], in the present study goal setting and follow-up support activities were less often provided during CHD care. Furthermore, key elements of the CCM, namely assisting patients with self management and arranging follow-up support, were provided significantly less often, indicating possible quality deficiencies and areas for quality improvement, particularly in relation to continuity of care [40].

The second and third findings are related to factors associated with patients’ assessments of receiving structured chronic care and counselling. At the patient level, being male, having more frequent practice contacts and having fewer other conditions were associated with higher PACIC scores. Other studies reported higher PACIC scores for younger patients [41] or did not demonstrate significant associations with patients’ characteristics [16,19]. We applied the PACIC instrument to routine primary care settings in various European health care systems. Within these settings, patients who had been included in quality assessment previously scored significantly higher. This finding resonates with previous research, which demonstrated that patients who were enrolled in interventions and were cared for more intensively scored higher, especially when interventions were tailored to special elements of the CCM. For example, patients participating in disease-management programs have been reported as having higher PACIC scores than those receiving usual care [21]. Furthermore, PACIC scores of patients participating in patient-centred case-management interventions can be improved from baseline to post-interventional measurement [22]. These findings are consistent with the necessity to align evaluation research with care improvement strategies [42,43].

At the practice level, there was a positive association between patients’ evaluations of the quality of care they received and quality scores reflecting quality-management and cardiovascular-care processes of general practices. However, the variance proportion at this level was less than the variance proportion at patient or country level. The variance caused by country specific factors can only be explained marginally in this study, as explanatory variables at the country level were not available. Although the variance between practices relating to the patients’ evaluations was relatively small, practice quality scores explained a significant portion of these variance. It had been presumed that larger practices with more full time equivalent (FTE) GPs would provide good quality of care [44]. However, the number of FTE GPs was not associated with patients’ evaluations during this study.

There is not one standardised way in which to measure quality of care, and each method has strengths and limitations. Ideally, it would be desirable that patients’ evaluation of their experiences with health care were congruent to other quality measures of practice care, to provide feedback to health care providers. Previous research has demonstrated few associations between objective quality measures and patients’ satisfaction [32-34,45]. Wensing et al. stated that patients may assess care differently from recommended care strategies [45]. Furthermore, it has been argued that patients value humanistic and affective items (e.g. staff’s friendliness) more than items concerning organization and governance [46], and that patient assessments depend on the personal relationship to the practice team where trust, loyalty and positive regard may influence the assessment [34,47]. In addition, several processes and structures of care are outside the direct experience or observation of patients, and not all patients are capable of understanding the risks and benefits of clinical choices [35,48]. The quality of providers’ performance may not be reflected in patients’ perceptions if general satisfaction scores that summarize the assessment of different health care processes into one global score are used [34]. The lack of positive association between quality measurement from the patients’ perspective and other quality measures of practice performance may be due to different underlying theoretical constructs of various instruments being used to assess patient perspectives [42,49]. Patient satisfaction may reflect the relationship between patient and practice team, which is dependent on patient and practice characteristics, whereas patient experiences with care may focus on organisational and procedural aspects of care [33]. The PACIC instrument predominantly questions the receipt of specific clinical processes of care. The underlying theoretical construct of the PACIC is the CCM; a patient centred care approach that is proactive, planned and includes goal setting, problem-solving and follow-up support. Therefore, positive association of patients’ perspectives with practice quality measures can be explained, as the quality indicators of practice organisation and chronic care used during this study could be related to these proactive CCM elements [6]. As patient experience is an important component of quality of care, and patient involvement is central to achieving good outcomes, future research is necessary to specify the constructs of patient assessment instruments that are required to obtain valid and reliable patient judgements of health care processes, particularly if patient experience is used within pay for performance systems [43].

### Strengths and limitations

The EPA cardio study is one of the largest international studies concerning management of cardiovascular care in European primary care [36]. To eliminate different health care effects, countries with strong primary health care systems (UK, the Netherlands) and countries characterized by a weaker primary care orientation [50] were included, and multilevel modelling was used to adjust for these differences. We used validated patient measures and assessed practice quality indicators through well-proven means [24,25]. All measures were pilot tested before being included in this study [28].

However, in Germany, Austria and Switzerland it was difficult to enrol 36 practices per country, as intended in the study protocol. As the PACIC instrument was only validated in three languages, countries such as France and Slovenia were excluded, decreasing the number of eligible patients. As we used an observational design, it was not possible to demonstrate a causal relationship between included characteristics or measures and patients’ perceived quality of care.

## Conclusions

The results of this study demonstrate that from the perspective of patients, the delivery of structured chronic care and counselling could be improved in general practices, particularly the arrangement of follow-up contacts. This indicates possible quality deficiencies in the continuity of care. Although patients’ perspective is an important component of the multidimensional nature of quality, evaluations based on patient experience should be adjusted according to relevant patient characteristics including the severity of illness and gender. Our findings indicate that the quality of practice management and care is positively reflected in patients’ assessment of receiving structured chronic care and counselling. This provides evidence for integrating patients’ evaluations into quality improvement strategies to identify quality deficiencies and to provide feedback to health care providers. However, there is an argument that instruments to assess quality of care from patients’ perspective and other quality measures of health care may not be generally congruent.

### Ethics committee approval

The corresponding author explicitly states that any necessary ethics committee approval was secured for the study reported. Names and locations of the approving ethics committees are listed below:

Austria: Med Uni Graz, Auenbruggerplatz 2, A-8036 Graz, Prof Peter Rehak

Belgium: Commissie Medische Ethiek UZ Leuven, Campus Gasthuisberg, Herestraat 49, B-3000 Leuven

Finland: Ethics committee of the Kuopio University Hospital, Kuopio, Finland

France: Ethic Commitee (Comié de Protection des Personnes Île-de-France V, Hôpital Saint-Antoine 184, rue du Faubourg Saint-Antoine 75012 Paris)

Germany: Ethics committee University Hospital: “Medizinische Fakultät Heidelberg“, Alte Glockengießerei 11/1, D-69115 Heidelberg (S-413/2007)

Netherlands: Ethics committee CMO Region Arnhem-Nijmegen

Slovenia: Slovenian national committee on medical ethics (No 87/11/07)

Switzerland: KEK - Kantonale Ethikkommission, Bern

United Kingdom: North West Research Ethics Committee: 07/H1010/83

## Endnotes

^1^http://www.topaseurope.eu.

## Conflict of interests

The authors declare that they have no conflicts of interests.

## Authors' contributions

MW developed the overall outline of the EPA-Cardio project. SC co-coordinated the selection of performance indicators, on which the EPA-Cardio audit instrument is based. SL co-coordinated the development and pilot testing of the measures, carried out data analysis and wrote the draft and final version of this paper. JvL contributed to the development and selection of measures and the draft revisions. JR provided biometrical support and contributed to analyses. DO and TF contributed to analyses and methods. JS supervised the research reported in this paper. All authors critically assessed and approved this paper.

## Pre-publication history

The pre-publication history for this paper can be accessed here:

http://www.biomedcentral.com/1472-6963/12/221/prepub

## Supplementary Material

Additional file 1 Appendix - The PACIC 5A instrument *. Click here for file
